# The Tonsil in Tuberculosis

**Published:** 1898-07-02

**Authors:** 


### THE TONSIL IN TUBERCULOSIS.

It is always a matter of great interest to decide the route by which an infective disease enters the body, and especially so when it is found that the place of entry is somewhat different from what might have been expected to be the case. Nothing can be more natural than to imagine that such a disease as tuberculosis of the lungs, the infection of which is often admittedly carried by the air, should enter by way of the bronchial tubes, hence the great importance of investigations, the tendency of which is to show that, even in such an apparently straightforward case as this, the route traversed is sometimes much more circuitous. In a thesis on Latent Tuberculosis of the Tonsil, Dr. Hugh Walsham shows that, out of 34 consecutive cases in which the tonsils were examined post-mortem, they were found to be more or less tuberculous in 20. With two exceptions there was nothing during life in any of these cases to call attention to the tonsils, but it is, perhaps, worth bearing in mind that they all occurred at the City of London Hospital for Diseases of the Chest. This point is additionally interesting from the fact that the result of an examination of many examples of hypertrophied tonsils and adenoid vegetations removed during life, apparently not from "chest" cases, was entirely negative. Now comes the question, Was the implication of the tonsils which was demonstrated in the phthisical patients primary or secondary; was it the original place of entry, or were the tonsils infected by the tuberculous expectoration passing over them? To this Dr. Walsham answers that in some it was one, and in some the other. In the primary cases the route taken by the tuberculous virus would be as follows: The bacilli would first gain entrance into the human organism by way of the tonsil, where they would develop for a time; then they would spread by way of the lymphatics to the cervical and mediastinal glands, and so to the thoracic duct, the right side of the heart, and thus to the lungs. A striking case is given in illustration of this mode of access. In this case "the tuberculous ulcer at the back of the pharynx was very chronic in its course; in fact, there were appearances of healing at some parts. Microscopically also the sections show the fibroid character of the tubercle. The infection, starting thence from the pharynx, gradually spread to the cervical glands, which are found post mortem to be tuberculous. From here we may conceive that the bacilli may reach the thoracic duct and the right side of the heart, the pulmonary artery, and the lungs. Now, what do we find? Exactly what one would expect—a general shower of miliary tubercle all over both lungs. My post-mortem note on the lungs is as follows: 'Both lungs are stuffed from the apex to the base with grey miliary tubercle.' This case is, I think, clear and conclusive. The old tuberculous ulceration in the fauces, and the fresh grey miliary tubercle in the cervical glands and lungs lead one irresistibly to the conclusion that the primary source of infection was in the pharynx. There could be no question of the pharynx being infected by the passage over it of sputa laden with bacilli, for, as usually happens in these cases of miliary tuberculosis of the lungs, there was no sputum." Of course, there is nothing very new in all this, but the illustrative cases are admirable, and by proving the large number of instances in which tubercle is discovered, not merely in the crypts of the tonsils, but in the adenoid substance of the glands, we have a demonstration of what has hitherto perhaps been more of the nature of a working hypothesis. We are, however, somewhat surprised to find that in the bibliography which is appended to the article, as it appears in the *Lancet*, no mention is made of the work of Professor Allbutt and Mr. Pridgin Teale on Scrofulous Neck, in which the "mucous lining of the pharynx" is pointed out as one of the starting points of the infection by which tuberculous enlargement of the cervical glands is produced.

At the Medical Congress in 1881, Professor Allbutt read a paper which, remembering the date at which the observations were made, and the remarkable advances which have since been made in our knowledge of tuberculosis, is an interesting forecast of the position taken up at the present time. Glandular enlargements are, he says, "bubonic in origin," and the "most effective source of irritation by far is the mucous membrane of the pharynx." The nasal and pharyngeal passages are "the gates of the lungs, and over them the air passes depositing its particles as it goes." "By a beautiful mechanism . . . . these membranes are constantly passing this silt" away, but let there be a trifling delay or languor of the epithelial function "vitally irritant" particles may embed themselves on the "soft absorbent vesture of the throat." The mucous membrane "may overpower the evil, as strong turnips outgrow the fly; or it may fail to do so," in which case these vital irritants may "generate materials" which find their way into the lymphatics. The same process again takes place in the glands, and thus "after the original local cause may have ceased to operate . . . . secondary mischief may abide within, and become a new source of centrifugal mischief." He also points out that when these bubonic enlargements are neglected, they may lead to phthisis "by absorption of the partially voided caseous products," or, as it is put in the lectures on "Scrofulous Neck," one of the risks of a continuance of the local maladies is "an inoculation of the system with elements which favour the development of more general tuberculosis." As a forecast of coming events, these papers by Professor Allbutt and Mr. Teale are fully worth referring to, especially as they formed the starting point of a line of treatment which is now almost universally adopted.

